# Identification of the QTL-allele System Underlying Two High-Throughput Physiological Traits in the Chinese Soybean Germplasm Population

**DOI:** 10.3389/fgene.2021.600444

**Published:** 2021-02-25

**Authors:** Lei Wang, Fangdong Liu, Xiaoshuai Hao, Wubin Wang, Guangnan Xing, Jingjing Luo, Guodong Zhou, Jianbo He, Junyi Gai

**Affiliations:** ^1^Soybean Research Institute, Nanjing Agricultural University, Nanjing, China; ^2^Plant Phenomics Research Center, Nanjing Agricultural University, Nanjing, China; ^3^MARA National Center for Soybean Improvement, Nanjing Agricultural University, Nanjing, China; ^4^MARA Key Laboratory of Biology and Genetic Improvement of Soybean (General), Nanjing Agricultural University, Nanjing, China; ^5^State Key Laboratory for Crop Genetics and Germplasm Enhancement, Nanjing Agricultural University, Nanjing, China; ^6^Jiangsu Collaborative Innovation Center for Modern Crop Production, Nanjing Agricultural University, Nanjing, China

**Keywords:** annual wild soybean (G. soja Sieb. & Zucc.), cultivated soybean (G. max (L.) Merr.), chlorophyll index (CHL), high-throughput phenotyping, normalized difference vegetation index (NDVI), QTL-allele matrix, restricted two-stage multi-locus genome-wide association study (RTM-GWAS), spectral reflectance image

## Abstract

The QTL-allele system underlying two spectral reflectance physiological traits, NDVI (normalized difference vegetation index) and CHL (chlorophyll index), related to plant growth and yield was studied in the Chinese soybean germplasm population (CSGP), which consisted of 341 wild accessions (WA), farmer landraces (LR), and released cultivars (RC). Samples were evaluated in the Photosynthetic System II imaging platform at Nanjing Agricultural University. The NDVI and CHL data were obtained from hyperspectral reflectance images in a randomized incomplete block design experiment with two replicates. The NDVI and CHL ranged from 0.05–0.18 and 1.20–4.78, had averages of 0.11 and 3.57, and had heritabilities of 78.3% and 69.2%, respectively; the values of NDVI and CHL were both significantly higher in LR and RC than in WA. Using the RTM-GWAS (restricted two-stage multi-locus genome-wide association study) method, 38 and 32 QTLs with 89 and 82 alleles and 2–4 and 2–6 alleles per locus were identified for NDVI and CHL, respectively, which explained 48.36% and 51.35% of the phenotypic variation for NDVI and CHL, respectively. The QTL-allele matrices were established and separated into WA, LR, and RC submatrices. From WA to LR + RC, 4 alleles and 2 new loci emerged, and 1 allele was excluded for NDVI, whereas 6 alleles emerged, and no alleles were excluded, in LR + RC for CHL. Recombination was the major motivation of evolutionary differences. For NDVI and CHL, 39 and 32 candidate genes were annotated and assigned to GO groups, respectively, indicating a complex gene network. The NDVI and CHL were upstream traits that were relatively conservative in their genetic changes compared with those of downstream agronomic traits. High-throughput phenotyping integrated with RTM-GWAS provides an efficient procedure for studying the population genetics of traits.

## Introduction

With the rapid development of high-throughput genome sequencing technology, high-quality genotype data can be obtained quickly and cheaply, enabling the detection of quantitative trait loci (QTL) at a high level of resolution through genome-wide association studies (GWAS) (Shendure and Ji, 2008; Visscher et al., 2017). Previous QTL studies have primarily focused on the collection of phenotype data (phenotyping) for agronomic traits to achieve various breeding objectives. As upstream biological traits often underlie breeding target traits, there is much interest in identifying upstream traits for the control of downstream agronomic traits. These upstream traits generally consist of some physiological or biochemical traits that are time-consuming and difficult to identify without the appropriate tools. Both high-quality genotype and phenotype data are required for accurate and powerful QTL detection. Because of improvements in the reliability of current genotyping technologies, obtaining high-quality phenotype data in QTL studies has become a major challenge (Cobb et al., 2013). Recently, spectral reflectance has been developed as a high-throughput phenotyping technique (Rebetzke et al., 2019; Watt et al., 2020). Remote-sensing images have been widely used to measure crop traits, such as plant height, biomass, chlorophyll content, disease susceptibility, drought stress sensitivity, nitrogen content, and yield (Gitelson et al., 2003; Estrada et al., 2015; Nigon et al., 2015, Holman et al., 2016; Jay et al., 2017, Salas Fernandez et al., 2017; Pérez-Bueno et al., 2019). Specifically, the approach is based on quantifying differences in canopy spectral reflectance among varieties for the aforementioned traits (Yang et al., 2017). The high-throughput phenotyping platform usually consists of several sensors and automatic systems and provides an efficient method for characterizing plant phenotypes (Furbank and Tester, 2011).

Multispectral and hyperspectral reflectance images have been widely used in high-throughput phenotyping platforms; the spectral index has been found to be closely related to the growth and development of crops (Duan et al., 2017). In studies of hyperspectral remote-sensing technology, vegetation indices are typically used to maximize the relationship between certain reflectance wavelengths and plant function when the effect of background noise is controlled (Huete et al., 2002; Hatfield and Prueger, 2010). Most of the vegetation indices are correlated with plant parameters, such as pigment status, grain yield, NDVI (normalized difference vegetation index), RVI (ration vegetation index), and GNDVI (green and near-infrared difference vegetation index) (Wiegand et al., 1991; Peñuelas et al., 1997; Lewis et al., 1998; Rutkoski et al., 2016). NDVI is calculated based on the near-infrared spectrum and red-light spectrum (Tucker, 1979), which has been extensively used to evaluate crop growth and estimate nitrogen content, nitrogen uptake, and nitrogen efficiency in crops (Erdle et al., 2011; Samborski et al., 2015; Foster et al., 2017). Studies of crop diseases have also shown that NDVI can be used for crop disease assessment (Kumar et al., 2016). More recently, studies have shown that NDVI is closely related to crop yield (Hassan et al., 2019). Zhang et al. (2019) used hyperspectral remote sensing to establish plot-yield prediction models for field selection in large-scale soybean breeding programs. Specifically, they found that NDVI and RVI were the best combination of vegetation indices for plot-yield prediction in their models.

Chlorophyll is the primary component involved in plant photosynthesis and is closely related to biomass accumulation and yield formation, making it critically important for crop improvement. The rapid and non-destructive estimation of chlorophyll content facilitates genetic studies of chlorophyll. Chlorophyll content can be predicted using different wavelength spectra; for example, there is a strong correlation between the reflectance ratio of the near-infrared band to the 700-nm band and chlorophyll content (Gitelson et al., 2003). The hyperspectral sensor in the high-throughput phenotyping platform is often used to estimate the chlorophyll index (CHL), and this index has been widely used to evaluate chlorophyll content, crop biotic stress, and abiotic stress (Estrada et al., 2015; Awlia et al., 2016; Pérez-Bueno et al., 2019).

NDVI and CHL are both spectral reflectance physiological traits related to plant growth and yield. To evaluate the usefulness of these traits in breeding programs, knowledge of their variability and genetic basis in germplasms is essential. Previously, the measurement of these two physiological traits was tedious and often not possible using traditional instruments. Now, multispectral and hyperspectral images have greatly facilitated the measurement of these traits. The greenhouse high-throughput phenotyping platform (GHTPP) in the Plant Phenomics Research Center (PPRC) at Nanjing Agricultural University (NJAU) has been used by several studies. Phenotype data from the high-throughput phenotyping platforms of previous studies have primarily been used for the prediction of agronomic traits, such as plant yield (Maimaitijiang et al., 2020). However, there is a need for more studies to assess the genetic basis of high-throughput spectral reflectance phenotypes.

The aims of this study were the following: (i) characterize variation in two spectral reflectance physiological traits, NDVI and CHL, in the Chinese soybean germplasm population (CSGP), including wild accessions (WA), cultivated farmer landraces (LR), and released modern cultivars (RC), using the facilities and equipment of the GHTPP at the PPRC, NJAU, to compare wild and cultivated soybeans; (ii) explore genetic variation in QTL-alleles through association mapping using the novel RTM-GWAS procedure and evolutionary changes from WA to LR and RC; (iii) predict the genetic potentials of the germplasm population through recombination among the accessions; and (iv) predict the candidate genes as well as the gene constitutions of NDVI and CHL in the CSGP based on information in SoyBase^1^.

## Materials and Methods

### Plant Materials and Experimental Design

A total of 341 soybean accessions of the CSGP, including 76 WAs, 83 LRs, and 182 RCs, were sampled in this study. A randomized incomplete block design experiment with two replicates was conducted for high-throughput phenotyping. Because of the space limitations of the high-throughput phenotyping platform, the accessions were randomly divided into two groups. Two replicates of the phenotyping experiment were performed for the first group of 172 accessions on September 9, 2019 and October 13, 2019, and for the second group of 169 accessions on November 20, 2019 and May 1, 2020. For each test, approximately 4∼5 viable seeds were selected from each accession and were planted in a plastic pot (Φ23 × H17 cm). The experimental soil was a 3:1 mixture of vermiculite and nutrient soil; one best soybean seedling remained in each pot on the seventh day after sowing. The temperature in the greenhouse was maintained between 25–33°C, and light was provided for 16 hours (06:00 to 22:00).

### High-Throughput Phenotyping

The greenhouse high-throughput phenotyping platform at the PPRC, NJAU, was used for phenotyping. The platform consisted of a planting area, irrigation area, and PSII (Photosynthetic System II) imaging room. An automatic and high-throughput transfer system was used to transfer plants from the planting/growing area to the imaging room. The PSII imaging room was equipped with a camera system (CropReporter, Phenovation B.V., Netherlands, https://www.phenovation.com/) with a CCD (charge-coupled device) camera, spectral LEDs (light-emitting diodes) for actinic treatment, an illuminated area of 70 cm × 70 cm, and a spectral range of 350–1000 nm. The spectral reflectance images were captured at six different wavelengths. From these images, the NDVI and CHL of the plant canopy for individual pots were estimated. According to CropReporter, the NDVI was calculated as (*R*_NIR_−*R*_red_)/(*R*_NIR_ + *R*_red_), and the CHL was calculated as $R_{700}^{-1}-R_{\text{NIR}}^{-1}$, where *R*_NIR_, *R*_red_, and *R*_700_ are the spectral reflectance in the near-infrared band, the visible red band, and the 700-nm wavelength band, respectively.

The platform was also equipped with the automatic experiment management software IS Agriware Logistics (Version2018.06.99, Indigo Logistics, Netherlands) to control system operation, CropReporter (Version 4.4.2, Phenovation B.V., Netherlands) to control the camera system, and the image analysis software Data Analysis (Version 5.4.8-64b, Phenovation B.V, Netherlands) to process the data. The two traits were unitless, as they represent relative values of reflectance. All of the NDVI and CHL values were obtained directly from the platform system.

The automatic phenotype measurements began on the sixth day after sowing (DAS6). Each pot with a plant was transferred from the planting area into the PSII imaging room to measure NDVI and CHL. Each pot was then returned to the planting/growing area. This phenotyping process was automatically executed every 3 days, and a total of nine measurements (DAS6, DAS9, DAS12, DAS15, DAS18, DAS21, DAS24, DAS27, and DAS30, which means the 6th, 9th,…, and 30th day counting from sowing, respectively) were taken throughout the experimental period for each accession type.

### SNP Genotyping and SNPLDB Assembly

The 341 soybean accessions were genotyped with RAD-seq (restriction site-associated DNA sequencing) in previous studies (He et al., 2017; Fu et al., 2020; Liu et al., 2020). A total of 145,558, 82,966, and 98,482 SNPs were recovered in these three studies, respectively, and the intersection of SNP data from different studies was taken and filtered with a minor allele frequency > 2% (each allele is present in at least six individuals). A total of 44,931 SNPs were obtained and used in the present study.

The RTM-GWAS (restricted two-stage multi-locus genome-wide association study) procedure (He et al., 2017) was used for QTL-allele detection in this study. With RTM-GWAS, a total of 11,716 multi-allelic SNPLDB markers were assembled based on the 44,931 genome-wide SNPs. The number of alleles of the SNPLDB markers ranged from 2 to 11 with an average of 3.1, enabling the detection of QTLs with up to 11 alleles per locus.

### Statistical Analysis

The experiment consisted of an incomplete block design. The plot values were adjusted using the block means according to the equal block mean assumption because the material set in each block was randomly selected; therefore, the entire experiment was treated as a completely randomized design with two replicates. The linear model for the adjusted dataset was *y*_*i*_ = μ + *g*_*i*_ + ε*_*i*_*, where *y*_*i*_ is the observed corrected phenotype of the *i*-th accession, μ is the population mean, *g*_*i*_ is the genotypic effect of the *i*-th accession, and ε*_*i*_* is the random error following a normal distribution with a mean of zero and variance of σ^2^. The analysis of variance of the corrected phenotype data was performed using the PROC GLM in SAS/STAT 9.4 (SAS Institute, Cary, NC), and variance components were estimated using PROC VARCOMP with the REML method. The trait heritability estimate was calculated as $h^{2}=\sigma_{g}^{2}/\left(\sigma_{g}^{2}+\sigma^{2}/r\right)$, where $\sigma_{g}^{2}$ is the genetic variance, and *r* is the number of replicates. This *h*^2^ is heritability in narrow sense because $\sigma_{g}^{2}$ contains only additive and additive by additive genetic variance in selfpollinated soybean germplasm population.

### Phenotype Data Selection

There were nine measurements (DAS6, DAS9, DAS12, DAS15, DAS18, DAS21, DAS24, DAS27, and DAS30) for each trait (NDVI or CHL) each accession. The trait heritability value was used to assess the goodness of the trait expression, and the measurement with a highest heritability value was chosen to represent the trait. Therefore, the variance components and heritability values were estimated based on analysis of variance for all the nine measurements, and the measurement with the highest trait heritability value was used for genome-wide association study.

### Restricted Two-Stage Multi-Locus Genome-Wide Association Study (RTM-GWAS)

The RTM-GWAS method was used for QTL-allele detection (He et al., 2017). Briefly, RTM-GWAS first involved the construction of multi-allelic SNPLDB (SNP linkage disequilibrium block) markers by grouping multiple adjacent and tightly linked SNPs through the LD-block partition. Second, the genetic similarity coefficient matrix based on SNPLDB markers was used to correct for population structure bias by incorporating its eigenvectors as model covariates. Finally, two-stage association analysis was conducted to detect QTLs and their corresponding multiple alleles based on a multi-locus multi-allele model. The linear model of RTM-GWAS in matrix form is **y** = **1**μ + **Wa** + **Xb** + **e**, where **y** is the phenotype, μ is the population mean, **W** is the eigenvector matrix representing the population structure, **X** is the design matrix of locus genotype, **a** and **b** are vectors of corresponding effects. At the first stage, **X** includes only a single SNPLDB marker for pre-selection. At the second stage, **X** includes multiple SNPLDB markers for multi-locus modeling. In the present study, at the first stage under the single locus model, 1,296 and 2,147 SNPLDBs were pre-selected from the 11,716 SNPLDBs for the second stage of stepwise regression association analysis under the multi-locus model for NDVI and CHL, respectively. As the RTM-GWAS was based on the multi-locus model having built-in control for the experiment-wise error rate, a normal significance level of 0.05 was used for QTL detection in this study.

### Prediction of the Genetic Potential of NDVI and CHL in the CSGP

To predict the recombination potential of the population, all possible single crosses among entire accessions, among subpopulation accessions, and between subpopulation accessions were simulated *in silico* (He et al., 2017). For each cross, 2,000 inbred lines were derived, and the phenotypes were predicted for each line according to the QTL-allele matrix. Finally, the recombination potential of each cross was assessed using the 99th percentiles of the predicted phenotype data.

### Annotation of Candidate Genes and GO Analysis of NDVI and CHL

According to SoyBase(see footnote 1), the candidate genes for NDVI and CHL were annotated from the identified QTLs. Next, the annotated candidate genes were subjected to gene ontology (GO) analysis using the Williams 82 genome version 1 (Wm82.a1.v1.1) as the reference genome. The candidate genes were searched within the interval (with a 50-kb flanking expansion) of the associated loci. In order to have a preliminary validation of the annotated candidate genes, the RNA Seq-Atlas project data set in SoyBase (see footnote 1) was downloaded and analyzed to assess the expression level of the annotated genes for NDVI and CHL.

## Results

### Phenotypic Variation of NDVI and CHL in the CSGP

Phenotype measurements for each accession were taken nine times on different days during growth (DAS6, DAS9, DAS12, and DAS30); the trait heritability at each measurement ranged between 16.0–78.3% and 25.4–69.2% for NDVI and CHL, respectively. The NDVI at DAS21 and CHL at DAS24 had the highest heritabilities and were thus examined in subsequent analyses.

The frequency distribution showed that the NDVI ranged from 0.05 to 0.18 with an average of 0.11 (Table 1). The entire population was separated into WA, LR, and RC subpopulations; the mean NDVI of the WA was relatively small (0.07) with values ranging from 0.05–0.13. The mean NDVI of LR and RC was 0.11 and 0.12, respectively, with values ranging from 0.06–0.18 and 0.06–0.17, respectively. The CHL frequency distribution of the entire population ranged from 1.20 to 4.78, with an average of 3.57. The CHL mean of WA was 3.08 and ranged from 1.20–4.49; the CHL mean of LR and RC was 3.68 and 3.72, respectively, and ranged from 2.73–4.78 and 2.67–4.50, respectively (Table 1).

**TABLE 1:** Frequency distribution of NDVI and CHL in the Chinese soybean germplasm population.

**Trait**	**Pop.**	**Midpoint and frequency**											***N***	**Mean**	**Range**	***h*^2^ (%)**
NDVI		0.06	0.07	0.08	0.09	0.10	0.11	0.12	0.13	0.14	0.15	≥0.16				
	Entire	22	34	35	26	34	27	53	48	26	22	14	341	0.11	0.05–0.18	78.3
	WA	19	26	18	7	2	1	2	1	0	0	0	76	0.07^a^	0.05–0.13	
	LR	2	3	8	6	8	8	17	13	6	6	6	83	0.11^b^	0.06–0.18	
	RC	1	5	9	13	24	18	34	34	20	16	8	182	0.12^b^	0.06–0.17	
CHL		≤2.50	2.70	2.90	3.10	3.30	3.50	3.70	3.90	4.10	4.30	≥4.50				
	Entire	4	16	22	22	46	50	69	60	30	13	5	337	3.57	1.20–4.78	69.2
	WA	4	13	19	11	13	6	5	1	0	1	1	74	3.08^a^	1.20–4.49	
	LR	0	1	2	4	13	16	20	13	6	5	3	83	3.68^b^	2.73–4.78	
	RC	0	2	1	7	20	28	44	46	24	7	1	180	3.72^b^	2.67–4.50	

*Pop.: Population. *N*: the number of accessions.NDVI: normalized difference vegetation index, measured on the 21st day after sowing; CHL: chlorophyll index, measured on the 24th day after sowing. *h*^2^, trait heritability.WA, wild accessions; LR, cultivated farmer landraces; RC, released modern cultivars.^*a,b*^different letters indicate significant differences between subpopulations based on *t*-tests at a significance level of 0.01.*

Both NDVI and CHL, two physiological traits related to photosynthesis and growth, significantly differed between wild (0.07, 3.08) and cultivated (0.11–0.12, 3.68–3.72) soybeans, suggesting that cultivated soybeans have experienced significant improvements in photosynthesis- and growth-related traits following their domestication (Table 1). Thus, these basic shortcomings of wild soybean should not be neglected when wild soybeans are used to improve cultivated soybeans.

The analysis of variance revealed significant differences among accessions for both NDVI and CHL, indicating that there was significant genetic variation for the two spectral reflectance traits (Supplementary Table 1). The trait heritability was estimated to be 78.3% for NDVI and 69.2% for CHL (Table 1). These findings indicate that phenotypic variation (PV) was primarily driven by genetic factors, and the underlying QTLs or genes could be traced through further genetic analysis.

### Identification of the QTL-allele System Determining NDVI and CHL in the CSGP

According to the RTM-GWAS procedure involving the use of 1,296 and 2,147 multi-allelic SNPLDB markers preselected at the first stage for the second stage multi-locus multi-allele association analysis, a total of 38 and 32 SNPLDBs, each with 2–4 and 2–6 alleles were determined to be significantly associated with NDVI and CHL, respectively (Table 2, Supplementary Tables 2, 4).

**TABLE 2:** The detected QTLs associated with NDVI and CHL in the CSGP.

**QTL**	**No. alleles**	**−lg*P***	***R*^2^ (%)**	**QTL**	**No. alleles**	**−lg*P***	***R*^2^ (%)**
*qNdvi-01-1*	2	3.80	1.28	*qChl-01-1*	3	7.32	2.97
*qNdvi-01-2*	2	1.82	0.53	*qChl-02-1*	2	8.17	2.96
*qNdvi-01-3*	3	4.06	1.69	*qChl-03-1*	2	3.66	1.18
*qNdvi-01-4*	2	8.42	3.18	*qChl-05-1*	2	2.98	0.93
*qNdvi-01-5*	2	1.72	0.49	*qChl-05-2*	2	1.96	0.56
*qNdvi-02-1*	4	8.33	3.84	*qChl-06-1*	2	2.37	0.70
*qNdvi-03-1*	2	2.85	0.92	*qChl-06-2*	3	6.74	2.73
*qNdvi-05-1*	2	1.96	0.58	*qChl-07-1*	3	3.01	1.20
*qNdvi-05-2*	2	1.46	0.40	*qChl-07-2*	3	6.35	2.57
*qNdvi-05-3*	4	7.73	3.57	*qChl-08-1*	2	1.84	0.51
*qNdvi-05-4*	4	3.80	1.82	*qChl-08-2*	3	12.97	5.38
*qNdvi-05-5*	2	1.74	0.50	*qChl-08-3*	3	3.74	1.50
*qNdvi-05-6*	2	1.55	0.43	*qChl-08-4*	2	1.46	0.38
*qNdvi-06-1*	2	6.24	2.27	*qChl-09-1*	2	2.41	0.72
*qNdvi-06-2*	2	2.44	0.76	*qChl-10-1*	3	12.42	5.14
*qNdvi-06-3*	2	2.58	0.81	*qChl-11-1*	3	5.90	2.38
*qNdvi-08-1*	2	2.73	0.87	*qChl-13-1*	2	2.59	0.78
*qNdvi-08-2*	2	3.96	1.35	*qChl-13-2*	2	3.93	1.29
*qNdvi-08-3*	2	3.36	1.11	*qChl-14-1*	2	1.91	0.54
*qNdvi-10-1*	3	1.78	0.74	*qChl-14-2*	2	2.77	0.85
*qNdvi-10-2*	2	5.58	2.00	*qChl-15-1*	2	1.68	0.46
*qNdvi-11-1*	2	2.58	0.81	*qChl-15-2*	2	2.50	0.75
*qNdvi-11-2*	2	5.16	1.83	*qChl-15-3*	2	1.98	0.56
*qNdvi-11-3*	3	2.18	0.90	*qChl-16-1*	2	4.24	1.40
*qNdvi-13-1*	3	2.57	1.06	*qChl-16-2*	2	3.66	1.18
*qNdvi-13-2*	2	1.98	0.59	*qChl-16-3*	2	3.78	1.23
*qNdvi-13-3*	2	1.37	0.37	*qChl-17-1*	2	2.24	0.66
*qNdvi-14-1*	2	1.63	0.46	*qChl-17-2*	2	5.88	2.04
*qNdvi-14-2*	3	7.06	2.97	*qChl-18-1*	4	2.65	1.26
*qNdvi-15-1*	3	1.89	0.78	*qChl-18-2*	4	3.58	1.66
*qNdvi-15-2*	3	6.27	2.63	*qChl-19-1*	6	4.25	2.36
*qNdvi-15-3*	2	3.88	1.32	*qChl-20-1*	4	5.59	2.52
*qNdvi-15-4*	2	2.06	0.62	**Total**	**82**	**32**	**51.35**
*qNdvi-16-1*	2	1.93	0.57				
*qNdvi-17-1*	2	2.63	0.83				
*qNdvi-18-1*	2	4.76	1.67				
*qNdvi-19-1*	2	3.53	1.18				
*qNdvi-19-2*	2	2.17	0.66				
**Total**	**89**	**38**	**48.36**				

*NDVI, normalized difference vegetation index, measured on the 21st day counting from sowing; CHL, chlorophyll index, measured on the 24th day counting from sowing; CSGP, Chinese soybean germplasm population. *h*^2^, trait heritability. *R*^2^, genetic contribution of a QTL; A QTL is designated as *qNdvi-01-1*, where -01 represents chromosome 1, and -1 represents its order on the chromosome; No. alleles: number of alleles in a SNPLDB; -lg*P, P*-value in the log10 scale of the association test in the RTM-GWAS. The corresponding SNPLDB of a QTL and its position are shown in Supplementary Table 2.*

**FIGURE 1:**
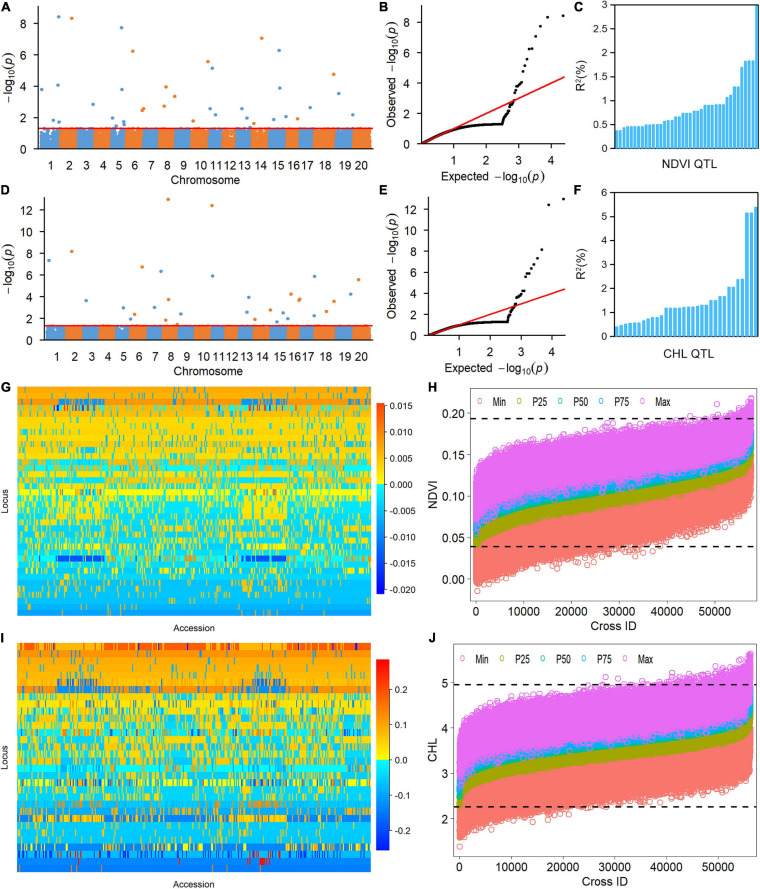
Genetic analysis of NDVI and CHL phenotypic variation in the soybean population using the restricted two-stage multi-locus genome-wide association study (RTM-GWAS) method. **(A)** Manhattan plot of the RTM-GWAS results for NDVI. **(B)** QQ plot of the RTM-GWAS results for NDVI. **(C)** The phenotypic contribution of the detected 38 NDVI QTLs. The vertical and horizontal axes indicate the genetic contribution *R*^2^ (%) and the order of QTLs according to their genetic contribution. **(D)** Manhattan plot of RTM-GWAS results for CHL. **(E)** QQ plot of the RTM-GWAS results for CHL. **(F)** The phenotypic contribution of the detected 32 CHL SNPLDBs. The vertical and horizontal axes indicate the genetic contribution *R*^2^ (%) and the order of QTLs according to their genetic contribution. **(G)** The NDVI QTL–allele matrix. **(H)** The predicted NDVI of progenies in the optimal crosses among the 341 lines based on the linkage model. On the horizontal axis, the crosses are arranged in increasing order of the predicted 50th percentile (P50) NDVI from left to right. The black dotted horizontal lines are the minimum and maximum values in the entire population, which were 0.039 and 0.193, respectively. The vertical axis is the predicted NDVI value of the crosses. **(I)** The CHL QTL–allele matrix. **(J)** The predicted CHL of the progenies in the optimal crosses among the 341 lines based on the linkage model. On the horizontal axis, the crosses are arranged in increasing order of the predicted 50th percentile (P50) NDVI from the left to right. The black dotted horizontal lines are the minimum and maximum values in the entire population, which were 2.26 and 4.95, respectively. The vertical axis is the predicted CHL value of the crosses.

The 38 NDVI-associated loci explained 48.39% of the phenotypic variation (PV), among which 17 large-contribution loci (*R*^2^ ≥ 1%) explained 34.77% of the PV and 21 small-contribution loci (*R*^2^ < 1%) explained 13.62% of the PV (Table 2). The phenotypic contribution of each associated locus to PV ranged between 0.37–3.84%. These loci were distributed on 15 chromosomes with 1 to 5 loci on each chromosome; chromosome 1 had the most loci (Figures 1A–C). In NDVI, the total genetic variation (heritability) was 78.3%, and the genetic contribution of the detected 38 QTLs was 48.39%; consequently, 29.91% of the genetic variation was not detected, which can be attributed to a collection of unmapped QTLs that needs to be further explored under controlled conditions where experimental error is minimized.

The CHL-associated loci had a similar pattern to those of NDVI (Table 2). The PV explained by the CHL-associated loci was 51.35%, among which 19 large-contribution loci explained 42.95% of the PV and 13 small-contribution loci explained 8.40% of the PV; the number of large-contribution loci was greater than the number of small-contribution loci. The PV of each associated locus ranged from 0.38 to 5.38%. These loci were distributed on 18 chromosomes with 1–6 loci on each chromosome; chromosome 5 had the most loci (Figures 1D–F). In CHL, the total genetic variation (trait heritability) was 69.2%, and the genetic contribution of the detected 32 QTLs was 51.35%; therefore, 27.85% of the genetic variation was not detected and will require further study to elucidate.

For other agronomic traits, such as 100-seed weight, days to flowering, and drought tolerance, the number of alleles per locus detected have been reported to range from 2–10 (He et al., 2017), 2–10 (Fu et al., 2020), and 2–12 (Wang et al., 2020), respectively. By comparison, the alleles per locus of NDVI and CHL in the present study were only 2–4 and 2–6, respectively, and the per-marker number of alleles was 2–11. The differences observed in these two spectral reflectance physiological traits potentially indicate that genetic differentiation at single loci is less likely for these two traits compared with other agronomic traits.

### QTL-allele Matrices of NDVI and CHL and Their Evolution From WA to LR and RC

**FIGURE 2:**
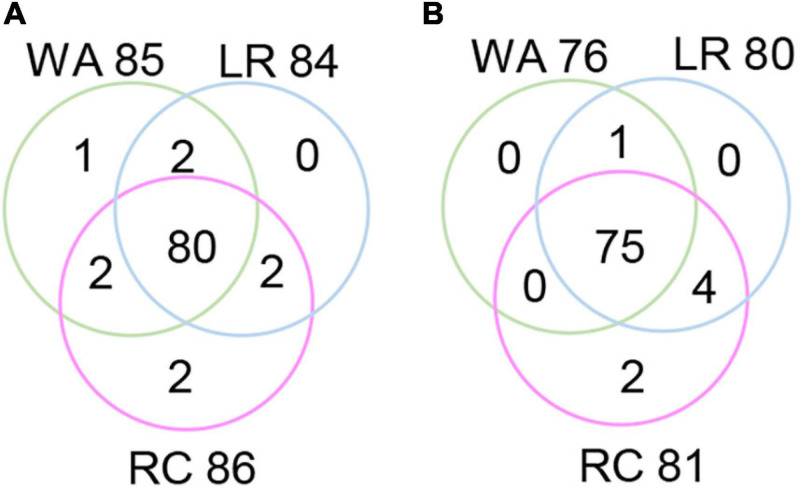
Venn diagram of QTL-allele changes among populations. **(A)** Venn diagram of the NDVI allele distribution in populations. **(B)** Venn diagram of the CHL allele distribution in populations. WA, wild accessions; LR, cultivated farmer landraces; RC, released modern cultivars.

**FIGURE 3:**
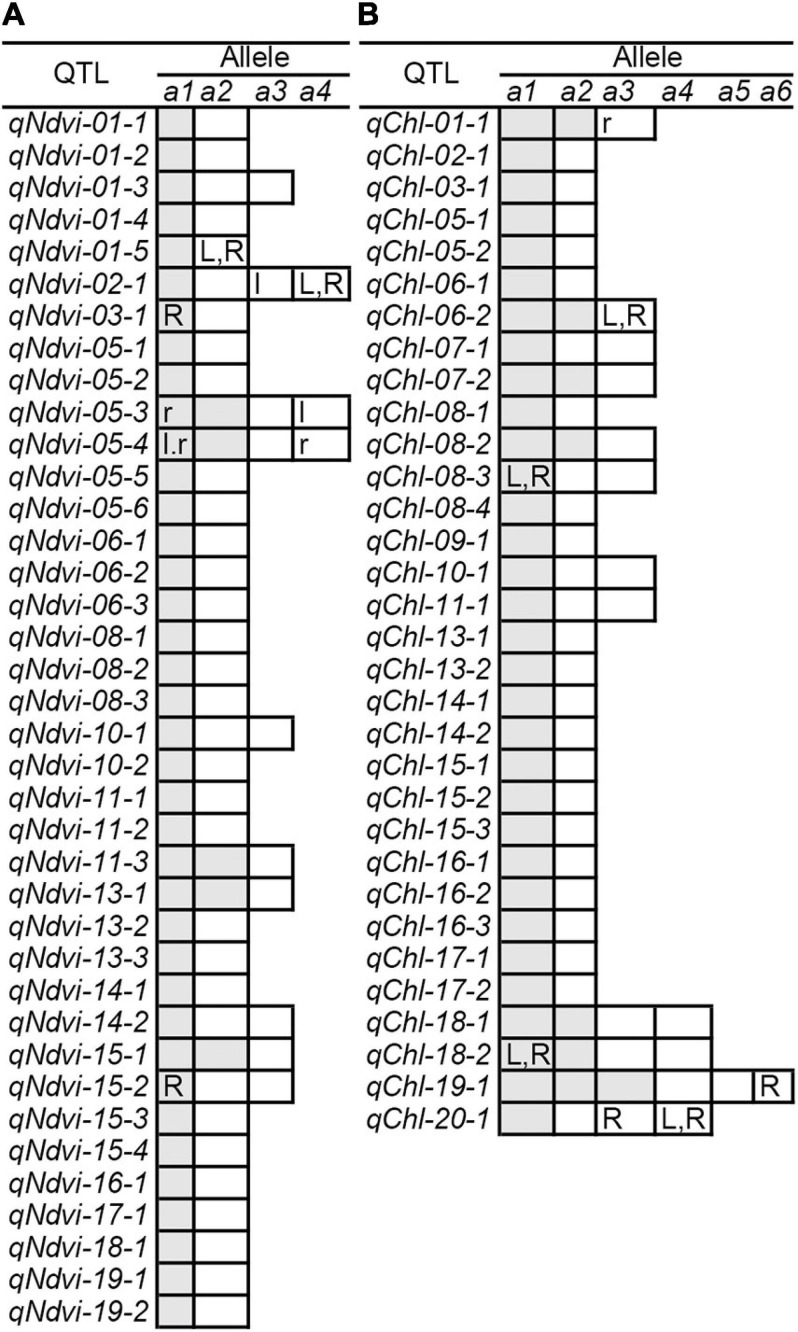
QTL-allele changes among populations. **(A)** NDVI QTL-allele changes among populations. **(B)** CHL QTL-allele changes among populations. *a1* –*a6* are the alleles of each QTL, arranged in descending order according to their allele effect values. The cells marked with white (positive effect) and gray (negative effect) are all alleles in WA. The cells with lowercase letters l and r are alleles excluded in LR and RC (vs. WA), respectively. The uppercase letters L and R in cells are alleles that emerged in LR and RC (vs. WA), respectively. In addition, the QTL *qNdvi-01-5* emerged in LR and was not polymorphic in WA. The QTL *qNdvi-03-1* emerged in RC and was not polymorphic in WA and LR.

The RTM-GWAS method provides a powerfull approach for the detection of genome-wide QTLs and their multiple allele effects. In this study, the effects of the 2–4 alleles per locus for a total of 89 alleles on 38 loci were obtained for NDVI. These QTL-alleles of the 341 accessions can be organized into a 38 × 341 (locus × accession) matrix (Figure 1G), which represents a compact form of the genetic structure of the population and was designated as the QTL-allele matrix of NDVI. Similarly, the effects of the 2–6 alleles per locus for a total of 82 alleles on 32 loci and the 32 × 341 (locus × accession) matrix were obtained for CHL (Figure 1I). The QTL-allele matrix detected from the RTM-GWAS contained all of the genetic constitutions of a trait in a population and can thus be used for the study of population genetic differentiation. The cultivated soybean is generally thought to have been domesticated from annual wild soybean, with released cultivars developed from farmer landraces (Liu et al., 2020). The QTL-allele matrix can be separated into its component matrices to facilitate the tracing of evolutionary genetic changes from WA to LR and RC. For NDVI, there were 85 alleles on 38 loci in WA; 82 wild alleles on 38 loci were passed to LR, with the emergence of 2 new alleles on 2 loci and the exclusion of 3 alleles on 3 loci for a total of 84 alleles on 38 loci (Table 3). From LR, 82 alleles on 38 loci were passed to RC, with the emergence of 2 new alleles on 2 loci, the recovery of 2 wild alleles on 2 loci, and the exclusion of 2 alleles on 2 loci for a total of 86 alleles on 38 loci. In LR + RC, 84 alleles on 38 loci were inherited from WA, including the emergence of 4 new alleles on 4 loci and the exclusion of 1 allele on 1 locus for a total of 88 alleles on 38 loci. Among the 4 newly emerged alleles on 4 loci in the cultivated LR + RC, 2 new loci with 2 new alleles emerged in LR + RC. In LR vs. WA, 1 of the 2 newly emerged alleles was in the newly formed QTL *qNdvi-01-5* in LR, which was not polymorphic in WA; in RC vs. LR, 1 of the 2 emerged alleles was in the newly formed QTL *qNdvi-03-1* in RC and was not polymorphic in WA and LR (Figures 2, 3; Table 3).

**TABLE 3:** The QTL-alleles changes from WA to LR and RC.

	**WA**		**LR vs. WA**		**RC vs. LR**		**LR + RC vs. WA**	
	**Locus**	**Allele**	**Locus**	**Allele**	**Locus**	**Allele**	**Locus**	**Allele**
**NDVI**								
Total	38	85	38	84	38	86	38	88
Inherited			38	82	38	82	38	84
Emerged			2	2	2	2	4	4
Recovery			–	–	2	2	–	–
Excluded			3	3	2	2	1	1
Changed			4	5	5	6	5	5
**CHL**								
Total	32	76	32	80	32	81	32	82
Inherited			32	76	32	80	32	76
Emerged			4	4	2	2	5	6
Recovery			–	–	–	–	–	–
Excluded			–	–	1	1	–	–
Changed			4	4	3	3	5	6

*WA, wild accession; LR, cultivated farmer landraces; RC, released modern cultivars. LR vs. WA means the number of changed alleles or changed loci of LR compared with WA. Inherited: the inherited allele/locus between populations. Emerged: the emerged allele/locus between populations. Excluded: the excluded allele/locus between populations. Changed: the changed allele/locus between populations (the emerged plus excluded alleles). Recovery: the alleles that were excluded from WA to LR but were recovered in RC. In LR vs. WA of NDVI, 1 of the 2 emerged alleles was in the newly formed QTL *qNdvi-01-5* in LR, which was not polymorphic in WA, while in RC vs. LR of NDVI, 1 of the 2 emerged alleles was in the newly formed. QTL *qNdvi-03-1* in RC, which was not polymorphic in WA and LR.*

Similar results were obtained for CHL. There were 76 alleles on 32 loci in WA; 76 wild alleles on 32 loci were passed to LR, with the emergence of 4 new alleles on 4 loci for a total of 80 alleles on 32 loci; no alleles were excluded (Table 3). From LR, 80 alleles on 32 loci were passed to RC, with the emergence of 2 new alleles on 2 loci and the exclusion of 1 allele on 1 locus for a total of 81 alleles on 32 loci; no wild alleles were recovered. In LR + RC, 76 alleles on 32 loci were inherited from WA, with the emergence of 6 new alleles on 5 loci for a total of 82 alleles on 32 loci; no wild alleles were excluded.

All of the emerged and excluded alleles and their associated QTLs are listed in Table 4. For NDVI, there were 4 newly emerged alleles on 4 loci (*qNdvi-01-5*, *qNdvi-02-1*, *qNdvi-03-1*, and *qNdvi-15-2*); *qNdvi-01-5* and *qNdvi-03-1* were also newly formed in LR and RC, respectively. One allele in *qNdvi-05-4* was excluded in LR, and 1 allele in *qNdvi-05-3* was excluded in RC. For CHL, there were 6 newly emerged alleles on 5 loci (*qChl-06-2, qChl-08-3, qChl-18-2, qChl-19-1, and qChl-20-1*), and 1 allele on *qChl-01-1* was excluded in RC. The allele frequencies of the newly emerged alleles in LR + RC ranged between 3.40–13.58%, and the new alleles were not dominant over older alleles.

**TABLE 4:** Emerged and excluded alleles conferring NDVI and CHL from WA to LR and then to RC.

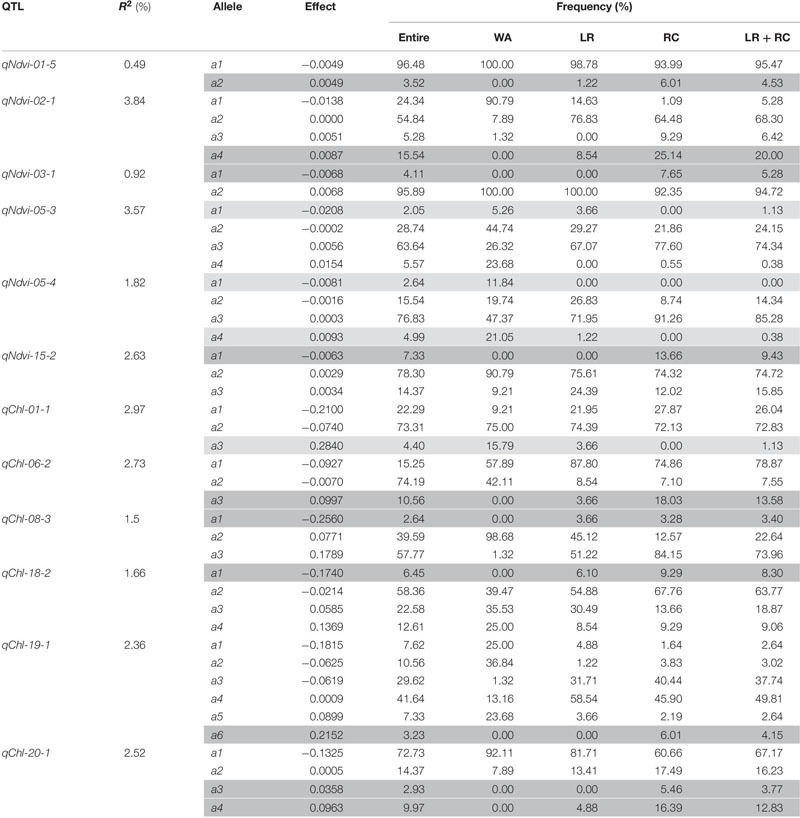

*WA, wild accession; LR, cultivated farmer landraces; RC, released modern cultivars. Boldface QTLs are newly emerged QTLs. Heavy shaded alleles are emerged ones, while light-shaded alleles are excluded ones; the number of changed alleles is consistent with that shown in Figure 2.*

Thus, genetic changes were limited during the evolution from WA to LR and RC, as the three subpopulations shared a large number of common alleles. Among the 89 wild alleles of 38 NDVI-associated loci, 80 alleles were shared among the three subpopulations and among the 76 wild alleles of 32 CHL-associated loci, and 75 wild alleles were shared among the three subpopulations (Figure 3). Here, the total change (emerged plus excluded) in alleles (5 alleles (5.7%) on 5 loci (13.2%) for NDVI and 6 alleles (7.3%) on 5 loci (15.6%) for CHL) was much lower relative to the changes in alleles observed for other agronomic traits. For example, there were a total of 261 alleles on 75 loci in Chinese cultivated soybeans for drought tolerance, and 46 alleles (17.6%) on 27 loci (36.0%) were changed in RC relative to LR (Wang et al., 2020). In addition, there were a total of 342 alleles on 81 loci in Northeast China soybeans for earliness, and 143 alleles (41.8%) on 67 loci (82.7%) were changed in the early group relative to the late group (Fu et al., 2020).

In summary, these two spectral reflectance physiological traits (NDVI and CHL) were genetically conservative. Inheritance played a major role in determining the genetic motivation, For NDVI, 4 alleles on 4 loci emerged; for CHL, 6 alleles on 5 loci emerged. Two new loci emerged for NDVI, but none emerged for CHL. For NDVI, only 1 allele on 1 locus was excluded, whereas no alleles were excluded for CHL. The transition from WA to LR and from LR to RC took approximately 5,000 and 100 years, respectively; despite this long history, large genetic changes have not occurred, especially during the transition from WA to LR. This genetic stability indicates that the two physiological traits NDVI and CHL are highly conservative compared with other agronomic traits. However, the same number of genetic changes occurred during the transition from WA to LR (5,000 years) and the transition from LR to RC (100 years), indicating that the enhanced artificial breeding in the transition from LR to RC accelerated the rate of genetic change.

### Prediction of the Recombination Potential of NDVI and CHL in the CSGP

To assess the recombination potential for NDVI and CHL in the CSGP, a total of 57,970 possible single crosses among the 341 accessions were simulated based on the QTL-allele matrix; possible crosses between the accessions for each subpopulation were also simulated. The 99th percentile of 2,000 progenies of each cross was used to represent the recombination potential (Table 5). For NDVI, the recombination potential within the WA, LR, and RC was not large (0.15 vs. 0.13 of the extreme phenotype, 0.20 vs. 0.18 of the extreme phenotype, and 0.20 vs. 0.17 of the extreme phenotype, respectively), but the predicted value was larger in LR and RC (the superior subpopulations) than in WA. Among the three between-subpopulation crosses, the highest recombination potential was observed for LR × RC (0.21 vs. 0.19 in WA × LR and WA × RC); for the crosses at the entire population level, the NDVI was 0.21, which was the same as that observed for LR × RC (Table 5, Figure 1H).

**TABLE 5:** The predicted optimal crosses for NDVI and CHL in the different material groups.

**Trait**	**Population**	**Extreme phenotype phenotype**	**No. crosses**	**Predicted phenotype**	
				**Mean**	**Max**
NDVI	WA	0.05–0.13	2,851	0.10	0.15
	LR	0.06–0.18	3,322	0.14	0.20
	RC	0.06–0.17	16,427	0.14	0.20
	WA × LR	–	6,234	0.13	0.19
	WA × RC	–	13,834	0.14	0.19
	LR × RC	–	14,926	0.14	0.21
	Entire	0.05–0.18	57,594	0.14	0.21
CHL	WA	1.20–4.49	2,702	3.66	4.93
	LR	2.63–4.78	3,322	4.18	5.31
	RC	2.67–4.50	16,111	4.20	5.06
	WA × LR	–	6,070	3.97	5.41
	WA × RC	–	13,322	4.01	5.38
	LR × RC	–	14,762	4.21	5.21
	Entire	1.20–4.78	56,289	4.11	5.41

*WA, wild accession; LR, cultivated farmer landraces; RC, released modern cultivars. The extreme phenotype is the observed minimum and maximum phenotype in the population. The predicted phenotype is the 99th percentile of a cross in the group of crosses.*

For CHL, the recombination potentials within WA, LR, and RC were also not large (4.93 vs. 4.49 of the extreme phenotype, 5.31 vs. 4.78 of the extreme phenotype, and 5.06 vs. 4.50 of the extreme phenotype, respectively), but the predicted value was larger in LR and RC (the superior populations) than in WA. Among the three between-subpopulation crosses, the highest recombination potential was observed for WA × LR (5.41 vs. 5.38 and 5.21 in WA × RC and LR × RC, respectively); for the crosses at the entire population level, the CHL was 5.41, which was the same as that observed for WA × LR (Table 5, Figure 1J). Thus, crosses with the WA had a greater recombination potential for CHL, which was opposite to the pattern observed for NDVI.

### Annotation of Candidate Genes and GO Analysis of NDVI and CHL in the CSGP

From the detected QTLs, a total of 39 candidate genes were annotated on 20 NDVI-associated loci, and 32 candidate genes on 22 CHL-associated loci (Supplementary Table 3). Only 9 candidate genes were annotated on 7 large-contribution loci of NDVI, but most (21 out of 32) of the CHL-related candidate genes were annotated on 13 large-contribution loci of CHL. Gene ontology (GO) analysis revealed that these candidate genes for both NDVI and CHL can be classified into three categories: biological process, molecular function, and cellular component (Table 6, Supplementary Figure 1). In biological process, NDVI involved 14 of 15 function groups, and CHL involved 11 of 15 function groups with five group differences. In molecular function, NDVI and CHL both involved 4 of 5 groups with two group differences. In cellular component, both NDVI and CHL involved all of the 5 function groups (Table 5, Supplementary Figure 1). The candidate gene systems of NDVI and CHL both involved a similar set of genes, although their frequency distributions differed. The two genetic systems consisted of a series of genes involved in a complex gene network.

**TABLE 6:** The number of candidate genes enriched in different GO annotations for NDVI and CHL.

**GO category**	**GO group**	**NDVI**	**CHL**
Biological process	Biological regulation	11	10
	Cellular component biogenesis	13	3
	Cellular process	20	18
	Developmental process	12	4
	Localization	4	3
	Metabolic process	18	19
	Multicellular organismal process	11	4
	Multi-organism process	7	–
	Response to stimulus	–	10
	Regulation of biological process	11	–
	Reproduction	9	–
	Response to stimulus	14	–
	Others	5	4
Molecular function	Binding	20	15
	Catalytic activity	12	9
	Structural molecule activity	3	–
	Transcription regulator activity	–	3
	Others	2	2
Cellular component	Cell	34	6
	Membrane	8	21
	Organelle	27	7
	Protein-containing complex	5	3
	Others	5	3
Total candidate genes		39	32

The validation of these candidate genes are left for further studies, however, a preliminary verification was carried out using the transcriptome data set of RNA Seq-Atlas project in SoyBase (see footnote 1). The gene expression results (Supplementary Figure 2) showed that 31 out of the 39 annotated genes for NDVI were expressed in 14 soybean tissues, among which *Glyma05g23230* and *Glyma14g06630* showed especially high expression level. For CHL, there were 28 out of 32 annotated genes were expressed in 14 soybean tissues, and *Glyma08g10960* showed high expression level in young leaf and pod shell, indicating these identified candidate genes are possibly functional.

## Discussion

### Efficiency of High-Throughput Phenotyping Integrated With RTM-GWAS in Identifying NDVI and CHL QTL-allele Systems

This study presented a genetic analysis of two spectral reflectance traits, NDVI and CHL, using a high-throughput phenotyping platform. Both NDVI and CHL showed significant genetic variation in CSGP, indicating that the spectral reflectance phenotyping data can not only be used for predicting agronomic traits but also for dissecting their underlying genetic basis. In this study, 89 alleles on 38 loci for NDVI and 82 alleles on 32 loci for CHL were detected, and the RTM-GWAS method was used to characterize their allele effects. High-throughput phenotyping integrated with RTM-GWAS was an efficient method for identifying the QTL-allele systems for NDVI and CHL. However, only 48.36% and 51.35% of the PV for NDVI and CHL were explained by the detected loci, which is low compared with other agronomic traits, such as 100-seed weight (139 QTLs explained 98.2% of the PV with a heritability of 98.9%, He et al., 2017). Although the large-contribution QTLs have been identified, many small-contribution QTLs consisting of unmapped minor QTLs have yet to be identified according to the RTM-GWAS. This observation might stem from experimental error given that the heritability values were only 78.3% and 69.2% for NDVI and CHL, respectively. Such error might have decreased the sensitivity of our analysis to detect QTLs, leaving 29.91% and 27.85% of the genetic variation (presumably unmapped minor QTLs) undetected. In the present study, an incomplete block design was conducted to separate all accessions into two sets for two respective tests to circumvent the space limitations associated with the high-throughput phenotyping platform. Although we employed a method to make the environment uniform between the different tests, much room for improvement remains.

In the present study, the PV explained by individual QTL ranged between 0.37-3.84% for NDVI, and 0.38-5.38% for CHL. There were 17 and 19 large-contribution (R2 ≥ 1%) and 21 and 13 small-contribution (R2 < 1%) QTLs for NDVI and CHL, respectively. The PV in RTM-GWAS is relatively lower than that in single-locus model such as the mixed linear model method (Yu et al., 2006). In single-locus model, association test is performed for each locus individually, and the estimated contribution of a locus may be inflated obviously due to the correlations among neighboring loci (He et al., 2017). But in RTM-GWAS, multiple QTLs are jointly fitted in a multi-locus model and the estimated PV for each QTL is unbiased and the total PV is controlled within heritability value, therefore, the individual QTL in RTM-GWAS may look smaller than those from single locus model procedure.

The fact of many QTLs each with a smaller PV is a characteristic of a quantitative trait controlled by a large number of QTLs. Or as we understand, the total PV of a quantitative trait in fact is a projection of a large number of genes/QTLs with different but interrelated biological functions onto the trait plane in a specific population. The statistically estimated genetic effect or PV of a QTL is relative to and largely depends on the genetic background of the population. A same QTL may exhibit varying effects in different populations with different genetic background. For example, the PV of a QTL in a simple genetic background such as near-isogenic lines is much greater than that in a germplasm population. Therefore, the small-contribution QTL in a study may exhibit large effects in other populations with simple genetic background. In fact, the purpose of the RTM-GWAS method is to achieve a relatively thorough detection of whole-genome QTLs and their multiple alleles or the QTL-allele system rather than a few individual large PV QTLs. Thus for the genetic improvement of quantitative traits in plant breeding, background control and foreground control are both important. It is likely that breakthroughs can be achieved through increase of positive alleles and decrease of negative alleles among multiple loci, rather than through recombination between/among a few loci like in the qualitative trait situation.

### Genetic Conservativeness of NDVI and CHL, Their Improvement Potential and Implications to Breeding for Seed-Yield of Soybeans

Both phenotypic and genotypic analysis showed that the two spectral reflectance traits were genetically conservative in comparison to the agronomic traits, such as seed yield, 100 seed weight, days to flowering (He et al., 2017; Zhang et al., 2019; Fu et al., 2020). Because in the present results, (i) no significant phenotypic improvements were observed in RC, and trait values were low in LR and RC; (ii) there was a limited number of alleles per locus; (iii) there was a large number of shared wild alleles among WA, LR, and RC, few new alleles, and little exclusion of wild alleles; and (iv) the recombination potential was low. In other words, the two spectral reflectance traits, NDVI and CHL, were more conservative than other agronomic traits in their genetic changes. We suspect that these spectral reflectance traits are upstream traits, whereas the other agronomic traits are downstream traits, the upstream traits may be more conservative than the downstream traits due to more factors may influence the downstream traits. For example, NDVI and CHL are traits related to the biological process of photosynthesis or organic synthesis while the agronomic traits such as seed yield may relate to the biological processes of transportation and storage of organics in addition to organic synthesis. The fact that breeding generally acts on downstream traits more readily compared to upstream traits may explain why the latter was more conservative and with less phenotypic improvements. Thus, additional effort is needed to improve upstream traits, such as NDVI and CHL, which are involved in light interception, light function and therefore, in photosynthesis and organics production.

However, some potential for improvement in NDVI and CHL was observed from WA to LR + RC, although the improvement was small (Table 1). The genetic mechanism underlying the observed evolutionary improvements might be recombination among loci-alleles given that all of the wild alleles passed to LR + RC except one negative wild allele excluded; furthermore, new alleles did not make up a large proportion of the alleles, given that few new alleles emerged (Tables 3, 4). This point is supported by the optimal cross prediction that the recombination among the loci/accessions might result in transgressive progenies, i.e., approximately 13–16% of genetic progress for NDVI and CHL might be achieved through hybridization in the CSGP (Table 5).

The previous studies on high-throughput phenotypes in crops usually focused on predicting yield-related agronomic traits, such as plant height, biomass and seed yield (Holman et al., 2016; Salas Fernandez et al., 2017, Maimaitijiang et al., 2020). For example, our previous results showed that NDVI was selected as the best vegetation indices in the establishment of plot-yield prediction models in breeding programs of soybeans (Zhang et al., 2019). Here in the present study, genetic dissection of the two high-throughput physiological traits, NDVI and CHL, was performed based on the high-throughput phenotyping technique. As NDVI and CHL are upstream traits and agronomic traits are breeding-acted target traits, identifying the genetic system of upstream traits may help to understand the genetic mechanism of downstream targets and also may provide additional control of downstream targets. For example, it was reported that NDVI was also a proxy for drought-adaptive traits in durum wheat, and high-throughput data collection of NDVI with capable precision can facilitate the genetic dissection of drought-adaptive traits (Condorelli et al., 2018). Thus, in breeding programs, breeders can combine the selection for upstream traits using high-throughput phenotyping data and the selection for downstream traits using agronomic data to have both selected and improved, which might benefit the enhanced selection of the downstream traits. This explains the reason that we suggested in yield breeding programs to combine the selection before harvest using NDVI prediction models established from hyperspectral reflectance data and the selection of harvested yield to achieve an enhanced selection for genotypic yield (Zhang et al., 2019).

## Data Availability Statement

The datasets presented in this study can be found in online repositories. The names of the repository/repositories and accession number(s) can be found below: https://github.com/njau-sri/leiwang2020ndvichl.

## Author Contributions

JG designed the study. LW and FL performed the experiments. JH, LW, and FL performed data analysis. XH, WW, GX, JL, and GZ participated in the experiments and data collection. JH, LW, and JG drafted the manuscript. All authors reviewed and approved the manuscript.

## Conflict of Interest

The authors declare that the research was conducted in the absence of any commercial or financial relationships that could be construed as a potential conflict of interest.

## Footnotes

1 http://soybase.org

## References

[B1] Awlia M. Nigro A. Fajkus J. Schmoeckel S. M. Negrão S. Santelia D. (2016). High-throughput non-destructive phenotyping of traits that contribute to salinity tolerance in *Arabidopsis thaliana*. *Front. Plant Sci*. 7:1414. 10.3389/fpls.2016.01414 27733855 PMC5039194

[B2] Cobb J. N. Declerck G. Greenberg A. Clark R. McCouch S. (2013). Next-generation phenotyping: requirements and strategies for enhancing our understanding of genotype-phenotype relationships and its relevance to crop improvement. *Theor. Appl. Genet*. 126 867–887. 10.1007/s00122-013-2066-0 23471459 PMC3607725

[B3] Condorelli G. E. Maccaferri M. Newcomb M. Andrade-Sanchez P. White J. W. French A. N. (2018). Comparative aerial and ground based high throughput phenotyping for the genetic dissection of NDVI as a proxy for drought adaptive traits in durum wheat. *Front. Plant Sci*. 9:893. 10.3389/fpls.2018.00893 29997645 PMC6028805

[B4] Duan T. Chapman S. C. Guo Y. Zheng B. (2017). Dynamic monitoring of NDVI in wheat agronomy and breeding trials using an unmanned aerial vehicle. *Field Crops Res*. 210 71–80. 10.1016/j.fcr.2017.05.025

[B5] Erdle K. Mistele B. Schmidhalter U. (2011). Comparison of active and passive spectral sensors in discriminating biomass parameters and nitrogen status in wheat cultivars. *Field Crops Res*. 124 74–84. 10.1016/j.fcr.2011.06.007

[B6] Estrada F. Escobar A. Romero-Bravo S. González-Talice J. Poblete-Echeverría C. Caligari P. D. S. (2015). Fluorescence phenotyping in blueberry breeding for genotype selection under drought conditions, with or without heat stress. *Sci. Hortic*. 181 147–161. 10.1016/j.scienta.2014.11.004

[B7] Foster A. J. Kakani V. G. Mosali J. (2017). Estimation of bioenergy crop yield and N status by hyperspectral canopy reflectance and partial least square regression. *Precis. Agric*. 18 192–209. 10.1007/s11119-016-9455-8

[B8] Fu M. Wang Y. Ren H. Du W. Wang D. Bao R. (2020). Genetic dynamics of earlier maturity group emergence in south-to-north extension of Northeast China soybeans. *Theor. Appl. Genet*. 133 1839–1857. 32030467 10.1007/s00122-020-03558-4

[B9] Furbank R. T. Tester M. (2011). Phenomics—technologies to relieve the phenotyping bottleneck. *Trends Plant Sci*. 16 635–644. 10.1016/j.tplants.2011.09.005 22074787

[B10] Gitelson A. A. Gritz Y. Merzlyak M. N. (2003). Relationships between leaf chlorophyll content and spectral reflectance and algorithms for non-destructive chlorophyll assessment in higher plant leaves. *J. Plant Physiol*. 160 271–282. 10.1078/0176-1617-00887 12749084

[B11] Hassan M. A. Yang M. Rasheed A. Yang G. Reynolds M. Xia X. (2019). A rapid monitoring of NDVI across the wheat growth cycle for grain yield prediction using a multi-spectral UAV platform. *Plant Sci*. 282 95–103. 10.1016/j.plantsci.2018.10.022 31003615

[B12] Hatfield J. Prueger J. (2010). Value of using different vegetative indices to quantify agricultural crop characteristics at different growth stages under varying management practices. *Remote Sens*. 2 562–578. 10.3390/rs2020562

[B13] He J. Meng S. Zhao T. Xing G. Yang S. Li Y. (2017). An innovative procedure of genome-wide association analysis fits studies on germplasm population and plant breeding. *Theor. Appl. Genet*. 130 2327–2343. 10.1007/s00122-017-2962-9 28828506

[B14] Holman F. Riche A. Michalski A. Castle M. Wooster M. Hawkesford M. (2016). High throughput field phenotyping of wheat plant height and growth rate in field plot trials using UAV based remote sensing. *Remote Sens*. 8:1031. 10.3390/rs8121031

[B15] Huete A. Didan K. Miura T. Rodriguez E. Gao X. Ferreira L. (2002). Overview of the radiometric and biophysical performance of the MODIS vegetation indices. *Remote Sens. Environ*. 83 195–213. 10.1016/s0034-4257(02)00096-2

[B16] Jay S. Maupas F. Bendoula R. Gorretta N. (2017). Retrieving LAI, chlorophyll and nitrogen contents in sugar beet crops from multi-angular optical remote sensing: comparison of vegetation indices and PROSAIL inversion for field phenotyping. *Field Crops Res*. 210 33–46. 10.1016/j.fcr.2017.05.005

[B17] Kumar S. Röder M. S. Singh R. P. Kumar S. Chand R. Joshi A. K. (2016). Mapping of spot blotch disease resistance using NDVI as a substitute to visual observation in wheat (*Triticum aestivum* L.). *Mol. Breed*. 36:95. 10.1007/s11032-016-0515-6

[B18] Lewis J. Rowland J. Nadeau A. (1998). Estimating maize production in Kenya using NDVI: some statistical considerations. *Int. J. Remote Sens*. 19 2609–2617. 10.1080/014311698214677

[B19] Liu F. He J. Wang W. Xing G. Gai J. (2020). Bi-phenotypic trait may be conferred by multiple alleles in a germplasm population. *Front. Genet*. 11:559. 10.3389/fgene.2020.00559 32582292 PMC7283545

[B20] Maimaitijiang M. Sagan V. Sidike P. Hartling S. Esposito F. Fritschi F. B. (2020). Soybean yield prediction from UAV using multimodal data fusion and deep learning. *Remote Sens. Environ*. 237:111599. 10.1016/j.rse.2019.111599

[B21] Nigon T. Mulla D. Rosen C. Cohen Y. Alchanatis V. Knight J. (2015). Hyperspectral aerial imagery for detecting nitrogen stress in two potato cultivars. *Comput. Electron. Agric*. 112 36–46. 10.1016/j.compag.2014.12.018

[B22] Peñuelas J. Isla R. Filella I. Araus J. (1997). Visible and near infrared reflectance assessment of salinity effects on barley. *Crop Sci*. 37 198–202. 10.2135/cropsci1997.0011183X003700010033x

[B23] Pérez-Bueno M. L. Pineda M. Barón M. (2019). Phenotyping plant responses to biotic stress by chlorophyll fluorescence imaging. *Front. Plant Sci*. 10:1135. 10.3389/fpls.2019.01135 31620158 PMC6759674

[B24] Rebetzke G. J. Jimenez-Berni J. Fischer R. A. Deery D. M. Smith D. J. (2019). Review: High-throughput phenotyping to enhance the use of crop genetic resources. *Plant Sci*. 282 40–48. 10.1016/j.plantsci.2018.06.017 31003610

[B25] Rutkoski J. Poland J. Mondal S. Autrique E. Pérez L. G. Crossa J. (2016). Canopy temperature and vegetation indices from high-throughput phenotyping improve accuracy of pedigree and genomic selection for grain yield in wheat. *G3 (Bethesda)* 6 2799–2808. 10.1534/g3.116.032888 27402362 PMC5015937

[B26] Salas Fernandez M. G. Bao Y. Tang L. Schnable P. S. (2017). A high-throughput, field-based phenotyping technology for tall biomass crops. *Plant Physiol*. 174 2008–2022. 10.1104/pp.17.00707 28620124 PMC5543940

[B27] Samborski S. M. Gozdowski D. Walsh O. S. Lamb D. W. Stępień M. Gacek E. S. (2015). Winter wheat genotype effect on canopy reflectance: implications for using NDVI for in-season nitrogen topdressing recommendations. *Agron. J*. 107 2097–2106. 10.2134/agronj14.0323

[B28] Shendure J. Ji H. (2008). Next-generation DNA sequencing. *Nat. Biotechnol*. 26 1135–1145. 10.1038/nbt1486 18846087

[B29] Tucker C. J. (1979). Red and photographic infrared linear combinations for monitoring vegetation. *Remote Sens. Environ*. 8 127–150. 10.1016/0034-4257(79)90013-0

[B30] Visscher P. M. Wray N. R. Zhang Q. Sklar P. McCarthy M. I. Brown M. A. (2017). 10 years of gwas discovery: biology, function, and translation. *Am. J. Hum. Genet*. 101 5–22. 10.1016/j.ajhg.2017.06.005 28686856 PMC5501872

[B31] Wang W. Zhou B. He J. Zhao J. Liu C. Chen X. (2020). Comprehensive identification of drought tolerance QTL-allele and candidate gene systems in Chinese cultivated soybean population. *Int. J. Mol. Sci*. 21:4830. 10.3390/ijms21144830 32650485 PMC7402128

[B32] Watt M. Fiorani F. Usadel B. Rascher U. Muller O. Schurr U. (2020). Phenotyping: new windows into the plant for breeders. *Annu. Rev. Plant Biol*. 71 689–712. 10.1146/annurev-arplant-042916-041124 32097567

[B33] Wiegand C. Richardson A. Escobar D. Gerbermann A. (1991). Vegetation indexes in crop assessment. *Remote Sens. Environ*. 35 105–119.

[B34] Yang G. Liu J. Zhao C. (2017). Unmanned aerial vehicle remote sensing for field-based crop phenotyping: current status and perspectives. *Front. Plant Sci*. 8:1111. 10.3389/fpls.2017.01111 28713402 PMC5492853

[B35] Yu J. Pressoir G. Briggs W. H. Vroh Bi I. Yamasaki M. Doebley J. F. (2006). A unified mixed-model method for association mapping that accounts for multiple levels of relatedness. *Nat. Genet*. 38 203–208. 10.1038/ng1702 16380716

[B36] Zhang X. Zhao J. Yang G. Liu J. Cao J. Li C. (2019). Establishment of plot-yield prediction models in soybean breeding programs using UAV-based hyperspectral remote sensing. *Remote Sens*. 11:2752. 10.3390/rs11232752

