# Supplementary material for: Identification of the QTL-allele System Underlying Two High-Throughput Physiological Traits in the Chinese Soybean Germplasm Population
Source: Front Genet. 2021 Feb 25;12:600444. doi: 10.3389/fgene.2021.600444 (PMC7947801; doi:10.3389/fgene.2021.600444)
Supplement: Supplementary file 1 [file Data_Sheet_1.docx]

***Supplementary Material***

Supplementary Tables

**Supplementary Table 1** Analysis of variance for NDVI and CHL

| Trait | Source of variation | DF | MS | *F* | *P* |
| --- | --- | --- | --- | --- | --- |
| NDVI | Accession | 339 | 0.52 | 4.41 | <0.001 |
|  | Error | 303 | 0.10 |  |  |
| CHL | Accession | 335 | 112.47 | 3.12 | <0.001 |
|  | Error | 294 | 31.59 |  |  |

NDVI: normalized difference vegetation index, measured on the 21st day after sowing; CHL: chlorophyll index, measured on the 24th days after sowing.

**Supplementary Table 2** The SNPLDBs and their positions of QTLs associated with NDVI and CHL

| QTL | SNPLDB | Position | QTL | SNPLDB | Position |
| --- | --- | --- | --- | --- | --- |
| *qNdvi-01-1* | Gm01_1528373 | 1528373 | *qChl-01-1* | Block_1_5116341 | 5116341-5116775 |
| *qNdvi-01-2* | Gm01_35308661 | 35308661 | *qChl-02-1* | Gm02_16336795 | 16336795 |
| *qNdvi-01-3* | Block_1_48524701 | 48524701-48529961 | *qChl-03-1* | Gm03_6682794 | 6682794 |
| *qNdvi-01-4* | Gm01_50195437 | 50195437 | *qChl-05-1* | Gm05_19851020 | 19851020 |
| *qNdvi-01-5* | Gm01_51802626 | 51802626 | *qChl-05-2* | Gm05_40997483 | 40997483 |
| *qNdvi-02-1* | Block_2_32054737 | 32054737-32140485 | *qChl-06-1* | Gm06_11834590 | 11834590 |
| *qNdvi-03-1* | Gm03_41960691 | 41960691 | *qChl-06-2* | Block_6_33761645 | 33761645-33761934 |
| *qNdvi-05-1* | Gm05_2355620 | 2355620 | *qChl-07-1* | Block_7_18965748 | 18965748-18967621 |
| *qNdvi-05-2* | Gm05_12374264 | 12374264 | *qChl-07-2* | Block_7_38448764 | 38448764-38456204 |
| *qNdvi-05-3* | Block_5_27675634 | 27675634-27733794 | *qChl-08-1* | Gm08_7996561 | 7996561 |
| *qNdvi-05-4* | Block_5_28810152 | 28810152-28840249 | *qChl-08-2* | Block_8_14777419 | 14777419-14795700 |
| *qNdvi-05-5* | Gm05_34860560 | 34860560 | *qChl-08-3* | Block_8_16373446 | 16373446-16420876 |
| *qNdvi-05-6* | Gm05_34953812 | 34953812 | *qChl-08-4* | Gm08_41623949 | 41623949 |
| *qNdvi-06-1* | Block_6_19277254 | 19277254-19277554 | *qChl-09-1* | Gm09_5765135 | 5765135 |
| *qNdvi-06-2* | Gm06_45944780 | 45944780 | *qChl-10-1* | Block_10_50033529 | 50033529-50074375 |
| *qNdvi-06-3* | Gm06_49654614 | 49654614 | *qChl-11-1* | Block_11_1086087 | 1086087-1086216 |
| *qNdvi-08-1* | Gm08_14753730 | 14753730 | *qChl-13-1* | Block_13_23133637 | 23133637-23152292 |
| *qNdvi-08-2* | Gm08_19702268 | 19702268 | *qChl-13-2* | Block_13_28533415 | 28533415-28666500 |
| *qNdvi-08-3* | Gm08_44817319 | 44817319 | *qChl-14-1* | Block14_2686637 | 2686637-2693043 |
| *qNdvi-10-1* | Block_10_3962423 | 3962423-3994205 | *qChl-14-2* | Gm14_47711114 | 47711114 |
| *qNdvi-10-2* | Gm10_46749398 | 46749398 | *qChl-15-1* | Gm15_17263902 | 17263902 |
| *qNdvi-11-1* | Gm11_1562066 | 1562066 | *qChl-15-2* | Gm15_35931319 | 35931319 |
| *qNdvi-11-2* | Block_11_8640579 | 8640579-8646084 | *qChl-15-3* | Gm15_49282552 | 49282552 |
| *qNdvi-11-3* | Block_11_17202479 | 17202479-17222849 | *qChl-16-1* | Gm16_7662261 | 7662261 |
| *qNdvi-13-1* | Block_13_14200897 | 14200897-14317831 | *qChl-16-2* | Block_16_31951721 | 31951721-31951751 |
| *qNdvi-13-2* | Gm13_29069366 | 29069366 | *qChl-16-3* | Gm16_33355539 | 33355539 |
| *qNdvi-13-3* | Gm13_38126326 | 38126326 | *qChl-17-1* | Gm17_38786884 | 38786884 |
| *qNdvi-14-1* | Gm14_4871116 | 4871116 | *qChl-17-2* | Gm17_40409578 | 40409578 |
| *qNdvi-14-2* | Block_14_27923435 | 27923435-27981962 | *qChl-18-1* | Block_18_33692262 | 33692262-33781931 |
| *qNdvi-15-1* | Block_15_12529854 | 12529854-12580045 | *qChl-18-2* | Block_18_55975160 | 55975160-55988446 |
| *qNdvi-15-2* | Block_15_27932357 | 27932357-27936943 | *qChl-19-1* | Block_19_42183676 | 42183676-42235298 |
| *qNdvi-15-3* | Gm15_30608859 | 30608859 | *qChl-20-1* | Block_20_15161057 | 15161057-15357939 |
| *qNdvi-15-4* | Gm15_47781916 | 47781916 |  |  |  |
| *qNdvi-16-1* | Gm16_30450973 | 30450973 |  |  |  |
| *qNdvi-17-1* | Gm17_29685800 | 29685800 |  |  |  |
| *qNdvi-18-1* | Gm18_55420833 | 55420833 |  |  |  |
| *qNdvi-19-1* | Gm19_8440427 | 8440427 |  |  |  |
| *qNdvi-19-2* | Gm19_48653597 | 48653597 |  |  |  |

NDVI: normalized difference vegetation index, measured on the 21st day counting from sowing; CHL: chlorophyll index, measured on the 24th day counting from sowing.

A QTL is designated as *qNdvi-01-1* where -01 represents chromosome 1, and -1 represents its order on the chromosome. The position corresponds to the Williams 82 reference genome version 1 (Wm82.a1).

**Supplementary Table 3** Annotated candidate genes conferring NDVI and CHL

| QTL | Gene | GO biological process | QTL | Gene | GO biological process |
| --- | --- | --- | --- | --- | --- |
| *qNdvi-01-1* | *Glyma01g01940* | mitochondrion | *qChl-03-1* | *Glyma03g06440* | translation |
|  | *Glyma01g01960* | protein transport |  | *Glyma03g06431* | unknown |
| *qNdvi-01-3* | *Glyma01g36070* | biosynthetic process | *qChl-05-2* | *Glyma05g37380* | protein glycosylation |
| *qNdvi-01-5* | *Glyma01g40010* | nitrate transport |  | *Glyma05g37390* | transcription factor |
|  | *Glyma01g40041* | unknown | *qChl-07-1* | *Glyma07g19040* | signal transduction |
| *qNdvi-03-1* | *Glyma03g34570* | proteolysis | *qChl-08-1* | *Glyma08g10960* | Rubber elongation factor protein |
|  | *Glyma03g34580* | Polyadenylate-binding protein | *qChl-08-2* | *Glyma08g19605* | arabinogalactan family protein |
|  | *Glyma03g34630* | cytoplasm | *qChl-08-3* | *Glyma08g21480* | cDNA |
| *qNdvi-05-1* | *Glyma05g03080* | protein binding |  | *Glyma08g21590* | cellular component |
|  | *Glyma05g03110* | mRNA catabolic process | *qChl-08-4* | *Glyma08g41670* | transducin family protein |
| *qNdvi-05-4* | *Glyma05g23230* | GTP catabolic process | *qChl-09-1* | *Glyma09g06890* | calcium ion transport |
| *qNdvi-05-5* | *Glyma05g29190* | protein phosphorylation | *qChl-10-1* | *Glyma10g43220* | regulation of transcription |
|  | *Glyma05g29250* | WD domain |  | *Glyma10g43280* | COP1-interacting protein |
|  | *Glyma05g29215* | leaf development | *qChl-11-1* | *Glyma11g01738* | protein phosphorylation |
| *qNdvi-05-6* | *Glyma05g29250* | WD domain | *qChl-13-1* | *Glyma13g19640* | nucleotide-sugar metabolic process |
| *qNdvi-08-3* | *Glyma08g45425* | cytokinesis |  | *Glyma13g19705* | molecular function |
| *qNdvi-10-1* | *Glyma10g05160* | mRNA catabolic process | *qChl-13-2* | *Glyma13g25310* | ATP binding |
|  | *Glyma10g05170* | phosphatidylinositol phosphorylation |  | *Glyma13g25440* | salicylic acid biosynthetic process |
|  | *Glyma10g05051* | seed dormancy | *qChl-14-1* | *Glyma14g04040* | las1-like family protein |
| *qNdvi-11-1* | *Glyma11g02460* | metabolic process | *qChl-14-2* | *Glyma14g38570* | Kinase interacting family protein |
| *qNdvi-11-2* | *Glyma11g12060* | regulation of transcription | *qChl-15-1* | *Glyma15g19820* | carbohydrate metabolic process |
|  | *Glyma11g12110* | DNA replication | *qChl-16-2* | *Glyma16g27980* | repeat family protein |
| *qNdvi-11-3* | *Glyma11g20390* | protein phosphorylation |  | *Glyma16g28080* | polysaccharide biosynthetic process |
|  | *Glyma11g20420* | floral organ formation | *qChl-16-3* | *Glyma16g29580* | nucleotide biosynthetic process |
|  | *Glyma11g20325* | chromatin silencing |  | *Glyma16g29630* | F-box family protein |
| *qNdvi-13-1* | *Glyma13g11650* | nucleotide binding | *qChl-17-1* | *Glyma17g34800* | dehydrase and lipid transport |
| *qNdvi-13-3* | *Glyma13g36940* | karyogamy | *qChl-17-2* | *Glyma17g36450* | chloroplast |
|  | *Glyma13g36970* | Putative N2 |  | *Glyma17g36490* | regulation of transcription |
| *qNdvi-14-1* | *Glyma14g06610* | intracellular protein transport | *qChl-18-1* | *Glyma18g29400* | regulation of transcription |
|  | *Glyma14g06630* | RNA methylation | *qChl-18-2* | *Glyma18g46220* | rRNA modification |
|  | *Glyma14g06640* | oxidative stress |  | *Glyma18g46270* | unknown |
|  | *Glyma14g06670* | innate immune response | *qChl-19-1* | *Glyma19g34630* | catabolic process |
|  | *Glyma14g06710* | transferase activity |  |  |  |
| *qNdvi-14-2* | *Glyma14g23590* | ARM repeat superfamily protein |  |  |  |
| *qNdvi-15-1* | *Glyma15g16320* | chloroplast stroma |  |  |  |
|  | *Glyma15g16202* | translation |  |  |  |
| *qNdvi-16-1* | *Glyma16g26311* | defense response |  |  |  |
| *qNdvi-19-2* | *Glyma19g42790* | cellular defense response |  |  |  |
|  | *Glyma19g42880* | biological process |  |  |  |

NDVI: normalized difference vegetation index, measured on the 21st day after sowing; CHL: chlorophyll index, measured on the 24th day after sowing.

**Supplementary Table 4** Allele effect and frequency of NDVI QTLs and CHL QTLs in CSGP

| QTL | Allele | Eff. | Freq. | QTL | Allele | Eff. | Freq. |
| --- | --- | --- | --- | --- | --- | --- | --- |
| *qNdvi-01-1* | *a1* | 0.00419 | 76.54 | *qChl-01-1* | *a1* | -0.074 | 73.31 |
|  | *a2* | -0.00419 | 23.46 |  | *a2* | -0.210 | 22.29 |
| *qNdvi-01-2* | *a1* | -0.00273 | 64.22 |  | *a3* | 0.284 | 4.40 |
|  | *a2* | 0.00273 | 35.78 | *qChl-02-1* | *a1* | 0.122 | 66.57 |
| *qNdvi-01-3* | *a1* | -0.00379 | 55.13 |  | *a2* | -0.122 | 33.43 |
|  | *a2* | 0.00314 | 23.46 | *qChl-03-1* | *a1* | 0.118 | 94.43 |
|  | *a3* | 0.00065 | 21.41 |  | *a2* | -0.118 | 5.57 |
| *qNdvi-01-4* | *a1* | 0.00256 | 75.37 | *qChl-05-1* | *a1* | -0.144 | 97.95 |
|  | *a2* | -0.00256 | 24.63 |  | *a2* | 0.144 | 2.05 |
| *qNdvi-01-5* | *a1* | -0.00491 | 96.48 | *qChl-05-2* | *a1* | 0.043 | 58.06 |
|  | *a2* | 0.00491 | 3.52 |  | *a2* | -0.043 | 41.94 |
| *qNdvi-02-1* | *a1* | 0.00001 | 54.84 | *qChl-06-1* | *a1* | -0.040 | 51.91 |
|  | *a2* | -0.01385 | 24.34 |  | *a2* | 0.040 | 48.09 |
|  | *a3* | 0.00873 | 15.54 | *qChl-06-2* | *a1* | -0.007 | 74.19 |
|  | *a4* | 0.00512 | 5.28 |  | *a2* | -0.093 | 15.25 |
| *qNdvi-03-1* | *a1* | 0.00683 | 95.89 |  | *a3* | 0.100 | 10.56 |
|  | *a2* | -0.00683 | 4.11 | *qChl-07-1* | *a1* | 0.029 | 78.30 |
| *qNdvi-05-1* | *a1* | -0.00461 | 86.80 |  | *a2* | -0.129 | 11.44 |
|  | *a2* | 0.00461 | 13.20 |  | *a3* | 0.101 | 10.26 |
| *qNdvi-05-2* | *a1* | -0.00328 | 90.62 | *qChl-07-2* | *a1* | -0.074 | 46.92 |
|  | *a2* | 0.00328 | 9.38 |  | *a2* | -0.053 | 36.66 |
| *qNdvi-05-3* | *a1* | 0.00562 | 63.64 |  | *a3* | 0.127 | 16.42 |
|  | *a2* | -0.00020 | 28.74 | *qChl-08-1* | *a1* | 0.045 | 53.67 |
|  | *a3* | 0.01541 | 5.57 |  | *a2* | -0.045 | 46.33 |
|  | *a4* | -0.02083 | 2.05 | *qChl-08-2* | *a1* | -0.133 | 67.74 |
| *qNdvi-05-4* | *a1* | 0.00031 | 76.83 |  | *a2* | -0.132 | 29.03 |
|  | *a2* | -0.00156 | 15.54 |  | *a3* | 0.265 | 3.23 |
|  | *a3* | 0.00931 | 4.99 | *qChl-08-3* | *a1* | 0.179 | 57.77 |
|  | *a4* | -0.00806 | 2.64 |  | *a2* | 0.077 | 39.59 |
| *qNdvi-05-5* | *a1* | -0.00288 | 83.87 |  | *a3* | -0.256 | 2.64 |
|  | *a2* | 0.00288 | 16.13 | *qChl-08-4* | *a1* | -0.060 | 90.03 |
| *qNdvi-05-6* | *a1* | -0.00266 | 72.73 |  | *a2* | 0.060 | 9.97 |
|  | *a2* | 0.00266 | 27.27 | *qChl-09-1* | *a1* | -0.048 | 75.95 |
| *qNdvi-06-1* | *a1* | 0.00843 | 75.66 |  | *a2* | 0.048 | 24.05 |
|  | *a2* | -0.00843 | 24.34 | *qChl-10-1* | *a1* | 0.064 | 52.20 |
| *qNdvi-06-2* | *a1* | -0.00286 | 63.93 |  | *a2* | 0.091 | 36.95 |
|  | *a2* | 0.00286 | 36.07 |  | *a3* | -0.155 | 10.85 |
| *qNdvi-06-3* | *a1* | 0.00620 | 92.67 | *qChl-11-1* | *a1* | -0.134 | 56.30 |
|  | *a2* | -0.00620 | 7.33 |  | *a2* | 0.089 | 22.58 |
| *qNdvi-08-1* | *a1* | 0.00266 | 57.77 |  | *a3* | 0.045 | 21.11 |
|  | *a2* | -0.00266 | 42.23 | *qChl-13-1* | *a1* | 0.057 | 56.30 |
| *qNdvi-08-2* | *a1* | -0.00341 | 63.64 |  | *a2* | -0.057 | 43.70 |
|  | *a2* | 0.00341 | 36.36 | *qChl-13-2* | *a1* | 0.092 | 96.77 |
| *qNdvi-08-3* | *a1* | -0.00466 | 87.39 |  | *a2* | -0.092 | 3.23 |
|  | *a2* | 0.00466 | 12.61 | *qChl-14-1* | *a1* | -0.053 | 69.50 |
| *qNdvi-10-1* | *a1* | -0.00422 | 70.97 |  | *a2* | 0.053 | 30.50 |
|  | *a2* | 0.00157 | 21.11 | *qChl-14-2* | *a1* | -0.058 | 90.62 |
|  | *a3* | 0.00265 | 7.92 |  | *a2* | 0.058 | 9.38 |
| *qNdvi-10-2* | *a1* | 0.00508 | 88.56 | *qChl-15-1* | *a1* | 0.078 | 90.62 |
|  | *a2* | -0.00508 | 11.44 |  | *a2* | -0.078 | 9.38 |
| *qNdvi-11-1* | *a1* | -0.00213 | 55.72 | *qChl-15-2* | *a1* | -0.047 | 78.01 |
|  | *a2* | 0.00213 | 44.28 |  | *a2* | 0.047 | 21.99 |
| *qNdvi-11-2* | *a1* | -0.00213 | 66.57 | *qChl-15-3* | *a1* | 0.048 | 73.02 |
|  | *a2* | 0.00213 | 33.43 |  | *a2* | -0.048 | 26.98 |
| *qNdvi-11-3* | *a1* | 0.00445 | 50.73 | *qChl-16-1* | *a1* | 0.059 | 51.32 |
|  | *a2* | -0.00231 | 28.74 |  | *a2* | -0.059 | 48.68 |
|  | *a3* | -0.00214 | 20.53 | *qChl-16-2* | *a1* | -0.088 | 73.90 |
| *qNdvi-13-1* | *a1* | -0.00040 | 62.46 |  | *a2* | 0.088 | 26.10 |
|  | *a2* | 0.00562 | 32.55 | *qChl-16-3* | *a1* | 0.083 | 93.84 |
|  | *a3* | -0.00522 | 4.99 |  | *a2* | -0.083 | 6.16 |
| *qNdvi-13-2* | *a1* | 0.00317 | 89.15 | *qChl-17-1* | *a1* | -0.061 | 89.74 |
|  | *a2* | -0.00317 | 10.85 |  | *a2* | 0.061 | 10.26 |
| *qNdvi-13-3* | *a1* | 0.00184 | 54.25 | *qChl-17-2* | *a1* | -0.062 | 53.08 |
|  | *a2* | -0.00184 | 45.75 |  | *a2* | 0.062 | 46.92 |
| *qNdvi-14-1* | *a1* | 0.00276 | 82.40 | *qChl-18-1* | *a1* | 0.035 | 57.77 |
|  | *a2* | -0.00276 | 17.60 |  | *a2* | -0.019 | 19.06 |
| *qNdvi-14-2* | *a1* | 0.00349 | 75.95 |  | *a3* | -0.096 | 14.96 |
|  | *a2* | -0.00420 | 19.06 |  | *a4* | 0.080 | 8.21 |
|  | *a3* | 0.00072 | 4.99 | *qChl-18-2* | *a1* | -0.021 | 58.36 |
| *qNdvi-15-1* | *a1* | 0.00543 | 47.80 |  | *a2* | 0.059 | 22.58 |
|  | *a2* | -0.00195 | 43.99 |  | *a3* | 0.137 | 12.61 |
|  | *a3* | -0.00348 | 8.21 |  | *a4* | -0.174 | 6.45 |
| *qNdvi-15-2* | *a1* | 0.00289 | 78.30 | *qChl-19-1* | *a1* | 0.001 | 41.64 |
|  | *a2* | 0.00340 | 14.37 |  | *a2* | -0.062 | 29.62 |
|  | *a3* | -0.00628 | 7.33 |  | *a3* | -0.063 | 10.56 |
| *qNdvi-15-3* | *a1* | -0.00610 | 96.48 |  | *a4* | -0.182 | 7.62 |
|  | *a2* | 0.00610 | 3.52 |  | *a5* | 0.090 | 7.33 |
| *qNdvi-15-4* | *a1* | -0.00720 | 96.77 |  | *a6* | 0.215 | 3.23 |
|  | *a2* | 0.00720 | 3.23 | *qChl-20-1* | *a1* | -0.133 | 72.73 |
| *qNdvi-16-1* | *a1* | -0.00264 | 78.59 |  | *a2* | 0.000 | 14.37 |
|  | *a2* | 0.00264 | 21.41 |  | *a3* | 0.096 | 9.97 |
| *qNdvi-17-1* | *a1* | 0.00324 | 94.43 |  | *a4* | 0.036 | 2.93 |
|  | *a2* | -0.00324 | 5.57 | 32 QTLs | 82 alleles |  |  |
| *qNdvi-18-1* | *a1* | -0.00343 | 64.81 |  |  |  |  |
|  | *a2* | 0.00343 | 35.19 |  |  |  |  |
| *qNdvi-19-1* | *a1* | -0.00495 | 82.99 |  |  |  |  |
|  | *a2* | 0.00495 | 17.01 |  |  |  |  |
| *qNdvi-19-2* | *a1* | 0.00337 | 86.51 |  |  |  |  |
|  | *a2* | -0.00337 | 13.49 |  |  |  |  |
| 38 QTLs | 89 alleles |  |  |  |  |  |  |

NDVI: normalized difference vegetation index, measured on the 21st day after sowing; CHL: chlorophyll index, measured on the 24th day after sowing. Eff.: allele effect of QTL. Freq.: allele frequency in CCSP. “*a1*” is the allele with a largest frequency on the QTL, while the others with their frequency in a descending order.


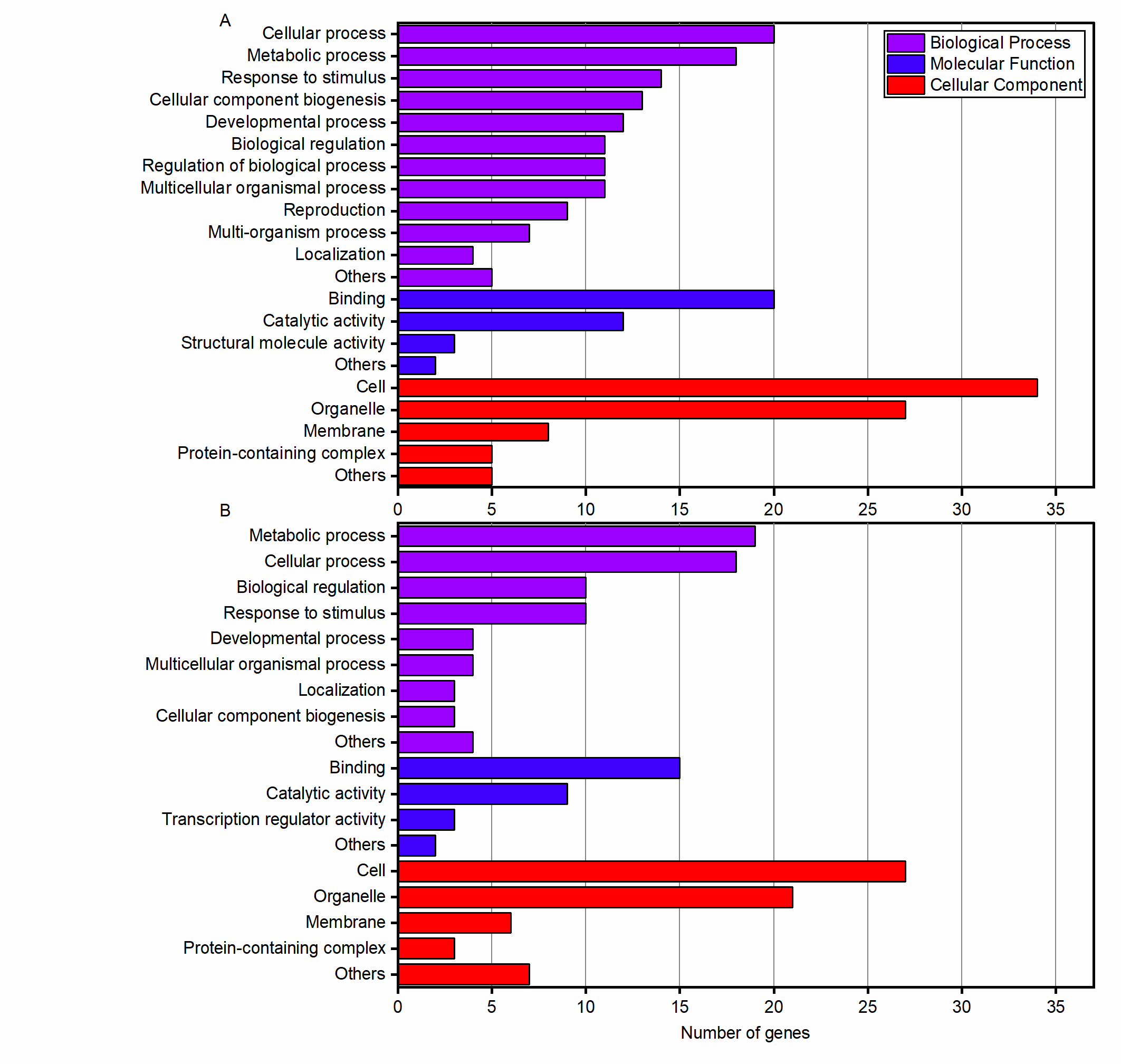


**Supplementary Figure 1** The GO analysis of candidate genes for NDVI (A) and CHL (B).

NDVI: normalized difference vegetation index; CHL: chlorophyll index.


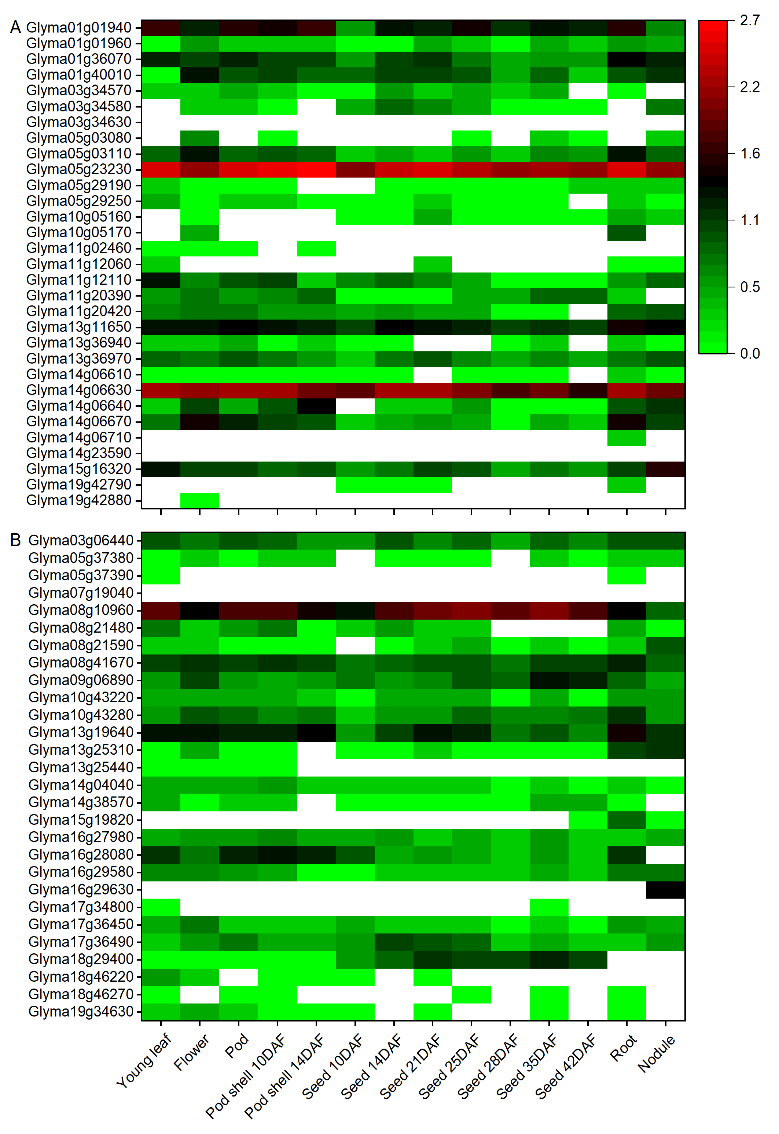


**Supplementary Figure 2** Digital expression profiles for NDVI (A) and CHL (B) in various tissues

NDVI: normalized difference vegetation index, measured on the 21st day after sowing; CHL: chlorophyll index, measured on the 24th day after sowing. Expression levels of annotated candidate genes in different vegetative tissues and at different seed developmental stages based on RNA sequencing data. The reads per kilobase million-normalized values were log_10_–transformed. The blank area means the expression level is 0. DAF is the days after flowering.
